# Giant Adrenal Pseudocysts: An Enigma for Surgeons

**DOI:** 10.1055/s-0042-1744153

**Published:** 2022-03-03

**Authors:** Kunal Parasar, Shantam Mohan, Aaron George John, Jitendra Nigam, Utpal Anand, Chandan Kumar Jha

**Affiliations:** 1 Department of Surgical Gastroenterology, All India Institute of Medical Sciences, Patna, Bihar, India; 2 Department of Gastroenterology, All India Institute of Medical Sciences, Patna, Bihar, India; 3 Department of Gastroenterology, Saroj Madan Gastro and Liver Clinic, Patna, Bihar, India; 4 Department of Pathology, All India Institute of Medical Sciences, Patna, Bihar, India; 5 Department of General Surgery, All India Institute of Medical Sciences, Patna, Bihar, India

**Keywords:** adrenal gland, neoplasms, adrenal pseudocyst, abdominal mass

## Abstract

Adrenal pseudocysts are cystic lesions arising within the adrenal gland enclosed by a fibrous connective tissue wall that lacks lining cells. They can attain a huge size and pose a diagnostic challenge with a broad range of differentials including benign and malignant neoplasms. There are only a few small case series and case reports describing these lesions. We report a series of five patients who presented with “indeterminate” abdominal cystic lesions and were later on found to have adrenal pseudocyst. Four out of five patients presented with non-specific abdominal symptoms, and one patient presented with symptoms suggestive of a functional adrenal tumor. The size of these tumors ranged from 6 to 30 cm. They had variable radiological features and in two cases even a percutaneous biopsy could not establish the diagnosis. In four of these “indeterminate” abdominal masses, an adrenal origin was not suspected preoperatively. Surgical excision provided a resolution of symptoms, ruled out malignancy, and clinched the diagnosis.

Adrenal pseudocysts are the most commonly reported cystic lesions of the adrenal gland in various surgical series. 1 2 Whether these cysts are a result of hemorrhage into non-neoplastic adrenal tissue, or they have a vascular origin is debatable, but there is little debate regarding the difficulties encountered in the preoperative diagnosis of these tumors. Most of these tumors are detected after they attain a large size and present with non-specific abdominal symptoms. The symptoms of hormonal excess, as present in the patients with other adrenal cortical or medullary lesions are classically absent in these tumors, hence its origin from the adrenal gland remains obscure on clinical evaluation. On abdominal imaging, their origin from the adrenal gland is easily missed because of the large size and variable imaging features. Even if an adrenal origin is suspected on imaging, differentiation from other adrenal tumors like adrenocortical carcinoma, adrenocortical adenoma, pheochromocytoma, and other cystic adrenal masses may not be possible. Similarly, image-guided FNAC/percutaneous biopsy has also been unreliable in arriving at a definitive diagnosis, although a distinction between benign and malignant masses can usually be made. 3 4 5 The presence of symptoms like pain and suspicion of malignancy are the usual indications for excision of these tumors which also allows the establishment of a definitive diagnosis for these tumors. We present our experience with adrenal pseudocysts in this retrospective study.

## Case Reports

The demographic, clinical, radiological, and follow-up details of the five cases are provided in Table 1 . Only one of our patients presented with symptoms of hormonal excess suggesting a pheochromocytoma, all others presented with non-specific symptoms of vague abdominal pain/discomfort or presence of an abdominal mass. After evaluation with contrast-enhanced computerized tomographic scan, a provisional diagnosis of hydatid cyst, retroperitoneal tumor with cystic degeneration, cystic vascular tumor, adrenocortical carcinoma, and a retroperitoneal tumor was suspected respectively in cases 1 to 5. Confirmation of the diagnosis with a percutaneous image-guided core needle biopsy was attempted in the two cases of suspected retroperitoneal tumors (cases 2 and 4), but both were inconclusive. In the other three cases, a biopsy was not attempted. We could not establish a diagnosis of pseudocyst in any of our five patients. In fact, the adrenal origin itself was not suspected in four out of five patients. The only case where an adrenal tumor was diagnosed preoperatively, underwent hormonal evaluation by estimation of plasma fractionated metanephrines and overnight dexamethasone suppression test (ONDST). The plasma metanephrines levels were normal and the ONDST was suppressible, precluding the diagnosis of a functional adrenal mass. Surgical excision was performed in all our cases because of the presence of symptoms/large size/suspicion of malignancy. No postoperative complications were observed. All were reported to be pseudocysts on histopathological examination. One patient died of an unrelated cause after 14 months of follow-up.

**Table 1:** Demographic, clinical, radiological, and follow-up details

	Case 1	Case 2	Case 3	Case 4	Case 5
Age in years	35	26	49	33	28
Gender	Female	Female	Female	Female	Male
Side	Right	Right	Left	Right	Right
Symptomatic	Yes	Yes	Yes	Yes	Yes
Pain	Yes	Yes	Yes	No	Yes
Abdominal mass	No	Yes	Yes	No	Yes
Symptoms of hormonal excess	No	No	No	Yes	No
Examination findings	None	Firm, irregular mass involving all quadrants	Firm cystic mass, variegated consistency, encroaching all quadrants	None	Firm mass with variegated consistency, involving all quadrants
CT features • Size • Margins • Solid component • Cystic component • Calcifications • Origin • Enhancement in solid component • Enhancement in cyst wall/septa • Septation • Hematoma • Layering • Others	• 10 cm • Well-defined, smooth • <25% • Septate cyst • No • Not clear • No • No • Yes • No • No • None	• 25 cm • Well-defined, smooth • Absent • Unilocular • No • Not clear • No • No • Yes • No • No • None	• 30 cm • Well-defined, smooth • >25% • Unilocular • Yes • Not clear • No • Yes (septa) • Yes • Yes • No • Dilated vessels on the surface	• 6 cm • Well-defined, smooth • Mostly solid • <25% • No • Right adrenal • Heterogenous • Yes • No • No • No • Dilated vessels on the surface	• 25 cm • Well-defined, smooth • Mostly solid • <25% • Yes • Not clear • Yes • No • No • No • No • Dilated vessels on the surface
Adrenal origin suspected	No	No	No	Yes	No
Plasma cortisol	Not done	Not done	Not done	WNL	Not done
Plasma metanephrines	Not done	Not done	Not done	WNL	Not done
FNAC/biopsy	Not recommended	Inconclusive	Not attempted	Not recommended	Inconclusive
Provisional diagnosis	Hydatid cyst of liver	Retroperitoneal tumor with cystic degeneration	Cystic vascular tumor	Adrenocortical carcinoma	Retroperitoneal sarcoma
Postoperative complication	None	None	None	None	None
Histology	Pseudocyst	Pseudocyst	Pseudocyst	Pseudocyst	Pseudocyst
Follow-up	Asymptomatic at 26 mo	Asymptomatic at 24 mo	Expired at 14 mo due to unrelated cause	Asymptomatic at 13 mo	Asymptomatic at 8 mo

## Discussion

Cystic lesions arising from the adrenal gland are encountered rarely. Autopsy series report an incidence between 0.064 and 0.18%. With improvements in imaging techniques, they are being detected more frequently and account for 1 to 22% of incidentally detected adrenal lesions. 6 Their size can vary from a few millimeters to more than 20 cm. They can be unilocular or multilocular. The majority of the cysts are unilateral but, rarely bilateral cysts have been reported. Incidence is higher in women than men. Incidence peaks between the third and sixth decade of life. 7 8

Adrenal cysts are classified into four types: pseudocysts, endothelial cysts, epithelial cysts, and parasitic cysts. 9 Adrenal pseudocysts are the most commonly reported in surgical series whereas endothelial cysts can account for up to 45% of adrenal cysts in autopsy series. 1 2 10

Adrenal pseudocysts are supposed to be the result of hemorrhage into non-neoplastic adrenal tissue. Theories postulate that it is of primarily vascular origin and immunohistochemical studies have supported this theory. Other theories postulate that pseudocysts represent the end-stage of lymphangiomatous lesions which undergo hemorrhage or degenerative change and its structure is replaced by fibrous tissue. The other postulated etiologies include changes in adrenal venous structures or blood vessel microvasculature. 11 12 A 7% risk of malignancy was noted in a review of 515 adrenal cysts, all of which were pseudocysts. 10 Pseudocysts arising from the adrenal cortex or medulla, are usually unilocular and filled with bloody or yellow–brown amorphous material. Pseudocysts are enclosed by a fibrous connective tissue wall that lacks lining cells. Histologically, the cyst wall is formed by hyalinized fibrous tissue of variable thickness and not lined by any demonstrable lining cells. The cysts' content may show an admixture of a variable amount of fibrin, fluid, and blood with or without macrophages, siderophages, and cholesterol clefts. Intracystic or intracapsular calcifications and islands and nests of normal-appearing adrenal cortical cells have also been reported. 9 11 12

**Fig. 1:**
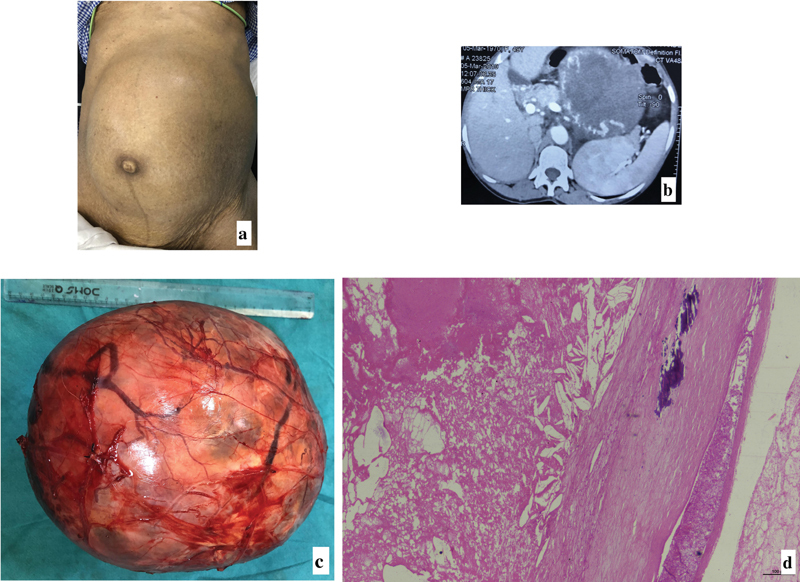
(Case 3) **(a)** Abdominal mass encroaching all quadrants; **(b)** CECT image showing a large, smooth, unilocular, predominantly cystic mass with enhancing solid areas and dilated vessels on the surface; **(c)** Excised specimen; **(d)** Cyst wall showing dystrophic calcification without any epithelial lining with compressed parenchyma on H&E stained sections (100x).

Small adrenal pseudocysts are usually incidentally detected on imaging of the abdomen. Large cysts cause a diagnostic dilemma and, in such cases, cystic masses that are likely to arise from surrounding structure must be considered in the differential. Renal cystic masses, which are much commoner constitute an important differential. 13 Other differentials to consider are other cystic adrenal masses, retroperitoneal tumors, hydatid disease of liver on the right side, and cystic pancreatic neoplasms on the left side. Larger adrenal pseudocysts may be symptomatic at the presentation which was the case in all our patients. 5 The symptoms usually are non-specific and related to the presence of a large abdominal mass. Symptom and/or signs of hormonal excess (Cushing's syndrome and/or hirsutism) if present is indicative of an underlying functional adrenocortical malignancy. The imaging findings of adrenal pseudocysts are highly variable; therefore, establishing a correct preoperative diagnosis is challenging even when a mass originating from an adrenal gland is apparent. The imaging appearance of the cyst varies from a completely cystic mass to a solid mass. The presence of septations, hypervascularity, and solid material (blood clots) causes heterogenicity within the cysts; this appearance can lead to a false diagnosis of malignant mass. 14 Calcification in the cyst wall is a common feature in the pure cystic form of the disease ( Fig. 1(b) ). The suggested radiographic criteria for diagnosing an adrenal cyst include a well-defined, sharply marginated mass of fluid attenuation without any evidence of enhancement. A fluid attenuation (>30 HU) may indicate hemorrhage, intracystic debris, or calcifications which are present in 15 to 70% of cases. 3 15 Because of the difficulty in coming to a definitive preoperative diagnosis due to its varied radiological presentations a cyst aspiration after ruling out pheochromocytoma or surgical excision is often needed to rule out malignancy. The risk of malignancy is high with cystic adrenal lesions that are heterogeneous, larger than 5 cm in size, thick-walled, or symptomatic. Such lesions warrant further evaluation, and surgical excision is a rational approach in them and remains the standard of care. 1

## Conclusion

Giant adrenal pseudocysts are rare lesions presenting between the third and sixth decade of life. They have a non-specific clinical presentation and variable radiological appearance. Surgical excision of large, symptomatic adrenal pseudocysts is required to rule out malignancy, arrive at a definitive diagnosis, and cure the patient.
